# Long-term cystatin C trajectories and cognitive decline in diabetes and non-diabetes: evidence from a national Chinese cohort

**DOI:** 10.3389/fmed.2026.1777079

**Published:** 2026-02-16

**Authors:** Xiangyang Zhou, Yuguang Zhao, Huaqiao Huo, Wenwen Cheng, Aiwen Feng, Shuang Wang

**Affiliations:** 1 Department of Neurosurgery, Maoming People’s Hospital, Maoming, China; 2 Department of Scientific Research Center, Maoming People’s Hospital, Maoming, China; 3 Department of Neurology, Maoming People’s Hospital, Maoming, China; 4 Department of General Surgery, Maoming People’s Hospital, Maoming, China

**Keywords:** cognitive decline, cumulative effects, cystatin C, glucose metabolism states, trajectory analysis

## Abstract

**Background:**

The relationship between Cystatin C levels and cognitive function remains unclear. This study aims to investigate the association between cumulative changes and trajectory patterns of Cystatin C and cognitive decline in diabetes and non-diabetes using a nationally representative cohort.

**Methods:**

A total of 3,733 participants from the China Health and Retirement Longitudinal Study (CHARLS) were included. Cumulative exposure was assessed based on repeated measurements from the first and third waves. K-means clustering was used to classify the longitudinal changes of Cystatin C. The long-term effects of Cystatin C on cognitive decline were evaluated, and subgroup analyses were performed in different populations. The mediating roles of glucose and HDL were also analyzed.

**Results:**

During the 3-year follow-up, cognitive decline occurred in 20.4% of the participants. In the non-DM (non-diabetes mellitus) group, cumulative Cystatin C increased the risk of cognitive decline. Compared with maintaining a medium level, maintaining a low level of Cystatin C was associated with a decreased risk of cognitive impairment. Subgroup analysis results showed that, in individuals with diabetes and aged ≥65 years, maintaining a low level of Cystatin C was associated with an increased risk of cognitive decline compared with maintaining a medium level. In non-diabetic individuals aged ≥65 years, cumulative Cystatin C increased the risk of cognitive decline. In non-diabetic and non-hypertensive individuals, maintaining a low level of Cystatin C was associated with a decreased risk of cognitive decline compared with maintaining a medium level.

**Conclusion:**

This study found that maintaining a low level of Cystatin C is associated with a lower risk of cognitive decline in non-diabetic individuals, while in older individuals with diabetes, maintaining a low level of Cystatin C increases the risk of cognitive decline. These findings suggest that the long-term dynamic changes in Cystatin C can serve as a useful indicator for assessing the risk of cognitive decline and highlight the need for differentiated preventive strategies in different populations.

## Introduction

Cognitive impairment is a leading cause of disability in older adults and has become a global public health priority in the context of population aging (1). Currently, more than 57 million people worldwide are living with dementia, and this number is projected to rise to over 150 million by 2050 (2). In China, 9.5 million individuals aged ≥60 years are affected by dementia (3). The high prevalence of cognitive impairment imposes a substantial burden on society and families.

Cystatin C (Cys C) is an endogenous cysteine protease inhibitor belonging to the type 2 cystatin superfamily and serves as an effective biomarker of renal function (4). The association between Cys C levels and cognitive function has been a subject of debate. Studies have shown that plasma Cys C levels are significantly higher in patients with dementia compared to healthy subjects (5). In patients with chronic kidney disease, elevated serum Cys C levels are associated with cognitive decline and increased likelihood of deficits in attention, executive function, and naming abilities (6). The Health ABC study demonstrated that higher levels of Cys C increase the risk of cognitive impairment (7). However, a 3-year prospective study indicated that Cys C acts as a protective factor against dementia progression in patients with mild cognitive impairment (8). A 10-year prospective study revealed a U-shaped relationship between Cys C concentration and the risk of cognitive impairment or dementia after 10 years, although this association disappeared after adjusting for confounding factors (9). Another study involving men aged 70–77 years showed that lower Cys C levels increased the risk of dementia, with a 0.1 μmol/L decrease in Cys C associated with a 29% increase in the risk of Alzheimer’s disease (10).

Diabetes is one of the most important cardiovascular risk factors. In 2021, the global number of adults aged 20–79 years with diabetes reached 536.6 million (an estimated prevalence of 10.5%). It is projected that by 2045, the number of people with diabetes will increase to 783.2 million (an estimated prevalence of 12.2%) (11). Diabetes can impair cognitive function through various pathophysiological mechanisms, such as amyloid-beta plaques, brain glucose metabolic disturbances, tau hyperphosphorylation, and inflammation (12–14). Studies have shown that cognitive decline in patients with type 2 diabetes (T2DM) is more severe than that in normal aging. Forty-five percent of T2DM patients experience cognitive dysfunction, with an increased risk of dementia (12). Older adults with T2DM are 50 to 100% more likely to develop dementia compared to those without T2DM (15).

Despite numerous studies investigating the association between Cys C and cognitive function, the results remain controversial. Previous studies have often focused on Cys C levels at a single time point, lacking long-term dynamic monitoring of Cys C levels. Moreover, research on the association between Cys C and cognitive function in diabetes and non-diabetes is limited.

Cumulative exposure and cluster trajectory are two recently proposed longitudinal data analysis methods that integrate repeated measurements (at two or more time points) and can better reflect the intensity and control level of exposure to a given parameter during follow-up. Therefore, this study aims to investigate the association between cumulative changes and trajectory patterns of Cys C and cognitive decline in diabetes and non-diabetes using a nationally representative cohort and longitudinal analysis methods, further elucidating the complex relationship between Cys C and cognitive function. This study integrates three methodological innovations: (i) stratification by glucose metabolic status (non-DM and DM); (ii) combined use of three-year cumulative exposure and k-means trajectory models to characterize Cystatin-C changes; (iii) formal testing of the mediating effects of fasting blood glucose and HDL-C. For the first time, the association between long-term dynamics of Cystatin-C and cognitive aging under different glucose metabolic backgrounds is elucidated at the population level.

## Methods

### Study design

This study utilized follow-up data from CHARLS, a national prospective cohort study conducted in China. The CHARLS national cohort was officially launched in 2011–2012, enrolling 17,708 participants from approximately 10,000 households across 450 village-level units in 150 counties of 28 provinces. The study aims to systematically collect and analyze data on the health and living conditions of the elderly population in China. Follow-up surveys are conducted every 2–3 years, and five surveys have been completed to date (2011–2012, 2013, 2015, 2018, and 2020). This continuous research approach ensures the timeliness and reliability of the data. Blood samples were successfully collected from participants during the first and third waves of the survey, and thus, this study analyzed the follow-up data from 2011 to 2012 and 2015. The CHARLS cohort has been approved by the Peking University Institutional Review Board (No. 00001052–11,015), and all participants provided written informed consent. The study was conducted in accordance with the principles of the Declaration of Helsinki.

Data from the CHARLS cohort between 2012 and 2015 were selected for this study. In 2012, there were 17,708 individuals at baseline. After excluding 8,439 individuals with missing cognitive scores in 2012/2015, 5,454 individuals with missing Cystatin C values in 2012/2015, and 82 individuals aged <45 years, a final sample of 3,733 participants was included in the study.

### Data collection

We collected various covariates from the CHARLS annual questionnaire, including demographic data (age, gender, education level, smoking and alcohol consumption status), basic measurements (height, weight, and waist circumference), medical history (hypertension, diabetes, and stroke), biochemical parameters, and cognitive scores. Height and weight were measured using standard methods with participants wearing light clothing and no shoes. Waist circumference (WC) was measured at the level of the umbilicus on the abdomen using a measuring tape while participants held their breath at the end of expiration. Body mass index (BMI) was calculated based on height and weight measurements.

Hypertension was clinically diagnosed when the mean systolic blood pressure was ≥140 mmHg and/or the mean diastolic blood pressure was ≥90 mmHg, or when participants were currently using antihypertensive medications. Diabetes was diagnosed based on self-reported physician diagnosis, fasting plasma glucose of 126 mg/dL or higher, or baseline glycated hemoglobin (HbA1c) of 6.5% or higher. History of stroke was determined by self-reported medical history. The non-diabetic group included all subjects except those with diabetes, including patients with prediabetes.

Venous blood samples were collected by trained professionals from the Chinese Center for Disease Control and Prevention in the morning after an overnight fast. To maintain the stability of the blood samples, all samples were stored at −80 °C to prevent loss or alteration of biochemical markers due to temperature fluctuations. Subsequently, trained laboratory personnel analyzed the venous blood samples using standard methods to measure biochemical indicators such as glucose, HDL-C, low-density lipoprotein cholesterol, total cholesterol, triglycerides (TG), and Cystatin C.

### Cumulative effect and trajectory analysis

**Figure 1:**
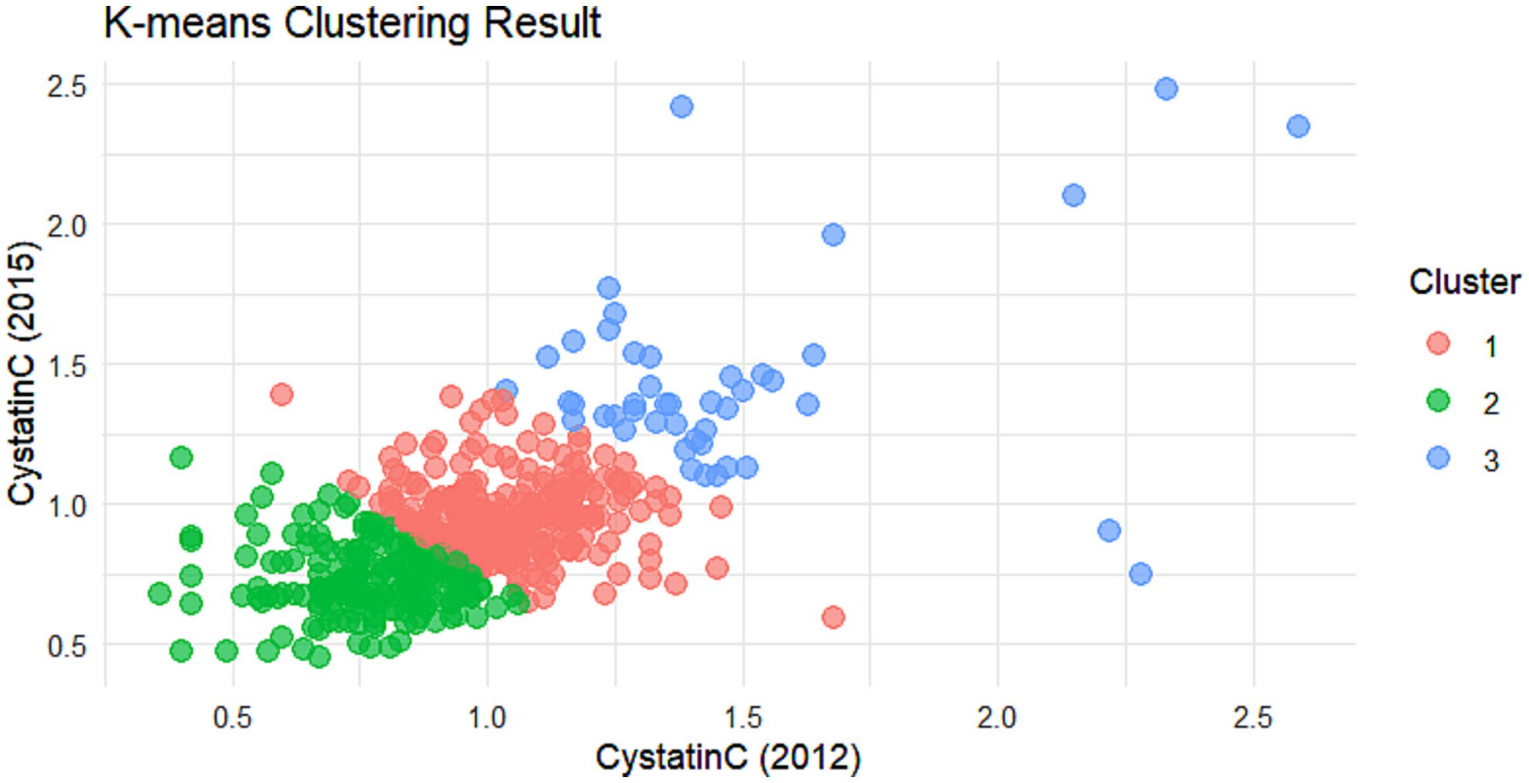
Cluster analysis dot plot for the DM group. Dot plot showing the distribution of cystatin C levels across different clusters in the DM group.

**Figure 2:**
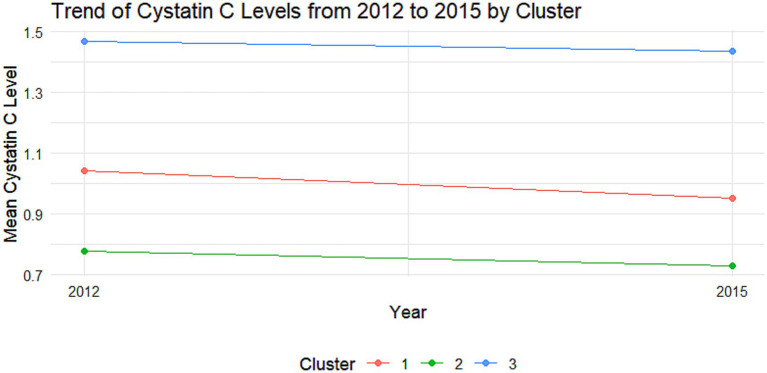
Trajectory analysis of cystatin C in the DM group. Trajectory plot illustrating the longitudinal changes in cystatin C levels over time in the DM group.

**Figure 3:**
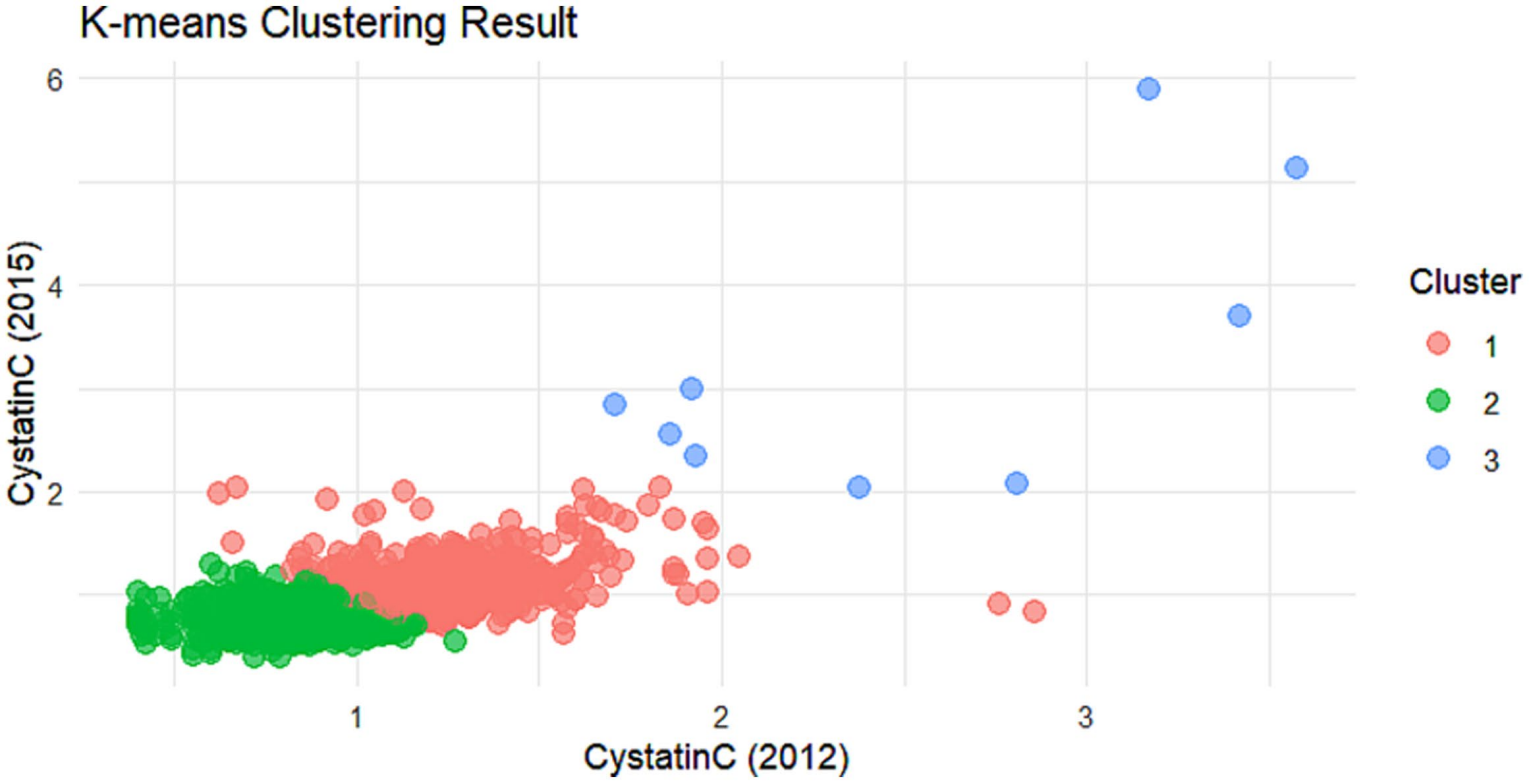
Cluster analysis dot plot for the non-DM group. Dot plot showing the distribution of Cystatin C levels across different clusters in the non-DM group.

**Figure 4:**
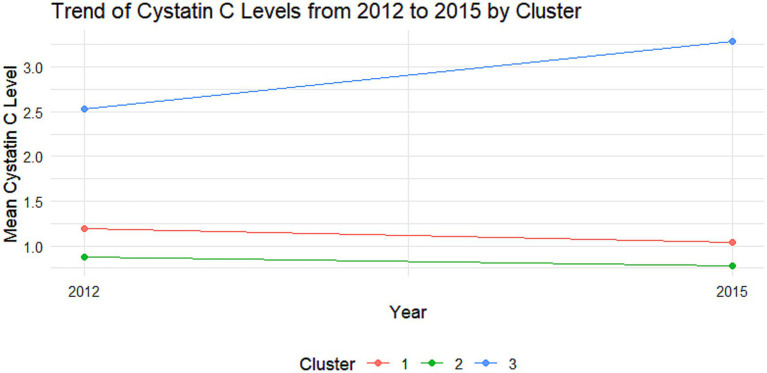
Trajectory analysis of Cystatin C in the non-DM group. Trajectory plot illustrating the longitudinal changes in Cystatin C levels over time in the non-DM group.

The cumulative effect of Cystatin C was calculated using the formula: (Cystatin C2012 + Cystatin C2015)/2 × time (2015–2012). All participants were followed up for 3 years. An incremental unit corresponds to a cumulative exposure equivalent to maintaining an average concentration 0.33 mg L^−1^ higher than the reference group for 3 years. The k-means clustering algorithm based on Euclidean distance was used to analyze the trajectories using baseline Cystatin C data and Cystatin C data from 2015. This algorithm offers high computational efficiency and intuitive visualization. We tested k = 2–5 using the elbow method and average silhouette width. The average silhouette coefficient peaked at k = 3 and produced clinically interpretable groups labeled Level 1, Level 2 and Level 3, k = 3 was adopted for all subsequent analyses. Based on the k-means clustering results, the diabetes group (Figures 1, 2) and the non-diabetes group (Figures 3, 4) were categorized as follows: Level 1: Stable at a medium level; Level 2: Stable at a low level; Level 3: Persistently high level. It is noteworthy that, due to the study only having two time points, this clustering is more of a pragmatic ‘pattern grouping’ rather than a multi-point trajectory model.

Supplementary Table 1 lists the sample sizes of each cluster in the DM and non-DM populations; Supplementary Table 2 provides the mean (centroid) values of Cys C for each cluster in 2012 and 2015 for intuitive comparison.

### Cognitive function assessment

In CHARLS, the cognitive function assessment score ranges from 0 to 21, with higher scores indicating better cognitive function. The assessment includes episodic memory (0–10 points) and mental integrity (0–11 points). Episodic memory was evaluated through a word recall test, including immediate and delayed recall, with the score being the average of the two recalls. Mental integrity was assessed using the Telephone Interview for Cognitive Status (TICS), which includes a serial 7 s subtraction test (0–5 points), date naming test (0–4 points), and a drawing task (0–1 point). Cognitive decline was defined as a decrease of 3 or more points in the cognitive score in 2015 compared to 2011–2012 (16).

### Quality assurance of cognitive assessment

Cognitive function was repeatedly assessed across Wave 1 to Wave 3 of the China Health and Retirement Longitudinal Study (CHARLS). The assessments were conducted face-to-face at baseline (Wave 1) and during follow-up waves (Wave 3) by trained interviewers in accordance with standardized CHARLS protocols. All interviewers received uniform, centralized training prior to data collection to ensure the consistency and reliability of cognitive test administration.

### Statistical analysis

Continuous variables are presented as mean (standard deviation), and categorical variables as counts (%). Between-group differences for continuous variables were compared using independent samples t-tests, while chi-square tests were used for categorical variables. Multivariate logistic regression analysis was performed to evaluate the multivariate associations between cumulative Cystatin C and Cystatin C trajectory categories with cognitive decline, with results expressed as odds ratio (OR) and 95% confidence intervals (CI). Restricted cubic spline (RCS) regression analysis was conducted using the rms package in R software to assess potential non-linear relationships between independent variables and outcomes. Mediation analysis between independent variables and cognitive decline was performed using R software packages. All analyses were completed using SPSS 25.0 and R software.

## Results

### Baseline characteristics of participants

A total of 3,733 participants were included in this study, comprising 1,937 males and 1796 females. The mean age was 58.90 years, with males averaging 60.07 years and females 57.65 years. The mean baseline Cystatin C level in 2012 was 0.99 mg/L, with males at 1.04 mg/L and females at 0.94 mg/L. By the end of the follow-up in 2015, the mean Cystatin C level was 0.88 mg/L, with males at 0.92 mg/L and females at 0.84 mg/L. The mean blood glucose level was 109.89 mg/dL, with a mean of 109.55 mg/dL in males and 5.28 mg/dL in females. The mean glycated hemoglobin was 5.26% overall, 5.23% in males and 5.28% in females. A total of 763 individuals experienced cognitive decline, including 386 males and 377 females (Table 1).

**Table 1:** Baseline characteristics of the participants.

Characteristic	Male	Female	Total
Age, years, mean (SD)	60.07 (8.66)	57.65 (8.52)	58.90 (8.68)
Education level			
Below primary school, n (%)	533 (27.5)	880 (49.1)	1,413 (37.9)
Primary school, n (%)	566 (29.3)	406 (22.6)	972 (26.1)
Junior high school, n (%)	550 (28.4)	340 (19.0)	890 (23.9)
Senior high school or above, n (%)	286 (14.8)	168 (9.4)	454 (12.2)
Smoking, n (%)	1,449 (74.8)	141 (7.9)	1,590 (42.6)
Alcohol consumption, n (%)	1,332 (68.8)	253 (14.1)	1,585 (42.5)
Hypertension, n (%)	856 (47.7)	896 (46.3)	1752 (47.0)
Diabetes mellitus, n (%)	270 (15.2)	270 (14.1)	540 (14.6)
Stroke, n (%)	44 (2.3)	31 (1.7)	75 (2.0)
BMI, kg/m^2^, mean (SD)	23.16 (3.59)	24.47 (4.01)	23.79 (3.85)
Waist circumference, cm, mean (SD)	84.68 (11.62)	85.76 (12.32)	85.20 (11.97)
WBC, ×10⁹/L, mean (SD)	6.40 (1.86)	6.01 (1.72)	6.21 (1.81)
Hematocrit, %, mean (SD)	43.76 (6.04)	39.67 (5.48)	41.79 (6.13)
PLT, ×10⁹/L, mean (SD)	202.81 (71.77)	220.30 (83.07)	211.21 (77.88)
Glu, mg/dL, mean (SD)	109.55 (34.92)	110.15 (34.43)	109.89 (34.68)
Glycated Hemoglobin, %	5.23 (0.73)	5.28 (0.81)	5.26 (0.77)
Cr, mg/dL, mean (SD)	0.87 (0.19)	0.69 (0.14)	0.79 (0.19)
TC, mg/dL, mean (SD)	188.61 (37.57)	198.45 (36.84)	193.35 (37.54)
TG, mg/dL, mean (SD)	128.50 (104.27)	141.34 (102.90)	134.68 (103.80)
HDL, mg/dL, mean (SD)	50.49 (16.08)	50.67 (14.03)	50.58 (15.13)
LDL, mg/dL, mean (SD)	113.07 (34.55)	120.53 (34.21)	116.66 (34.58)
Uric acid, mg/dL, mean (SD)	4.95 (1.26)	4.00 (1.04)	4.50 (1.25)
Cystatin C 2012, mg/L, mean (SD)	1.04 (0.24)	0.94 (0.22)	0.99 (0.23)
Cystatin C 2015, mg/L, mean (SD)	0.92 (0.27)	0.84 (0.21)	0.88 (0.24)
Cognitive decline, n (%)	386 (19.9)	377 (21.0)	763 (20.4)

### Univariate analysis of cognitive decline

Univariate analysis revealed that age, education level, hematocrit, HDL, baseline Cystatin C, Cystatin C in 2015, cumulative Cystatin C, and cumulative Cystatin C categories were associated with cognitive impairment (all *p* < 0.05). There was no significant difference in the glucose level and glycated hemoglobin levels between the two groups (*p* = 0.900). Specifically, older age, lower education level, lower hematocrit, higher HDL, higher Cystatin C levels, and higher cumulative Cystatin C levels were associated with the occurrence of cognitive decline (Table 2).

**Table 2:** Univariate analysis of factors associated with cognitive decline.

Characteristic	Non-cognitive decline group	Cognitive decline group	*p*
Gender:			0.421
Male, n (%)	1,551 (80.1)	386 (19.9)	
Female, n (%)	1,419 (47.8)	377 (21.0)	
Age, years, mean (SD)	58.36 (8.54)	61.02 (8.89)	<0.001
Education level:			<0.001
Below primary school, n (%)	1,019 (72.1)	394 (27.9)	
Primary school, n (%)	792 (81.5)	180 (18.5)	
Junior high school, n (%)	755 (84.8)	135 (15.2)	
Senior high school or above, n (%)	400 (88.1)	54 (15.2)	
Smoking, n (%)	1,256 (79.0)	334 (21.0)	0.442
Alcohol consumption, n (%)	1,284 (81.0)	301 (19.0)	0.059
Hypertension, n (%)	1,377 (78.6)	375 (21.4)	0.153
Diabetes mellitus, n (%)	421 (78.0)	119 (22.0)	0.322
Stroke, n (%)	55 (73.3)	20 (26.7)	0.175
BMI, kg/m^2^, mean (SD)	23.91 (3.91)	23.35 (3.81)	0.533
Waist circumference, cm, mean (SD)	85.38 (12.11)	84.51 (11.41)	0.088
WBC, ×10⁹/L, mean (SD)	6.19 (1.78)	6.22 (1.81)	0.722
Hematocrit, %, mean (SD)	41.91 (6.18)	41.35 (5.90)	0.025
PLT, ×10⁹/L, mean (SD)	213.45 (101.32)	210.63 (70.63)	0.473
Glu, mg/dL, mean (SD)	109.74 (34.31)	110.45 (36.14)	0.616
Glycated Hemoglobin, n (%)	5.25 (0.77)	5.26 (0.78)	0.900
Cr, mg/dL, mean (SD)	0.78 (0.19)	0.79 (0.20)	0.471
TC, mg/dL, mean (SD)	193.10 (37.26)	194.30 (38.62)	0.429
TG, mg/dL, mean (SD)	136.24 (106.47)	128.59 (92.56)	0.069
HDL, mg/dL, mean (SD)	50.25 (14.97)	51.87 (15.67)	0.008
LDL, mg/dL, mean (SD)	116.48 (34.29)	117.34 (35.72)	0.538
Uric acid, mg/dL, mean (SD)	4.49 (1.24)	4.51 (1.29)	0.762
Cystatin C 2012, mg/L, mean (SD)	0.98 (0.23)	1.02 (0.25)	<0.001
Cystatin C 2015, mg/L, mean (SD)	0.87 (0.22)	0.91 (0.30)	<0.001
Cumulative Cystatin C, mg/L, mean (SD)	2.78 (0.61)	2.90 (0.75)	<0.001
Cumulative Cystatin C groups:			<0.001
Q1, n (%)	759 (82.1)	165 (17.9)	
Q2, n (%)	788 (80.3)	193 (19.7)	
Q3, n (%)	718 (81.1)	167 (18.9)	
Q4, n (%)	705 (74.8)	238 (25.2)	

### Multivariate analysis of long-term cystatin C changes and cognitive decline

Multivariate analysis showed that, after adjusting for age, education level, hematocrit, and HDL, no significant associations were found between Cystatin C and its long-term changes and cognitive decline in the DM group (all *p* > 0.05). In the non-DM group, each one-unit increase in baseline Cystatin C was associated with a 60% increased risk of cognitive decline (OR = 1.60, 95% CI: 1.08–2.38, *p* = 0.020). Each one-unit increase in cumulative Cystatin C was associated with a 17% increased risk of cognitive decline (OR = 1.17, 95% CI: 1.02–1.35, *p* = 0.024). Compared to Cystatin C Level 1, Level 2 was associated with a 19% decreased risk of cognitive impairment (OR = 0.81, 95% CI: 0.66–0.99, *p* = 0.040; Table 3).

**Table 3:** Multivariate analysis of factors associated with cognitive decline*.

Characteristic	Reference	DM group		Non-DM group	
		OR (95% CI)	P	OR (95% CI)	*p*
Model 1					
Baseline Cystatin C, mg/L		0.50 (0.20, 1.25)	0.139	1.60 (1.08, 2.38)	0.020
Model 2					
Cumulative Cystatin C, mg/L		0.92 (0.66, 1.28)	0.601	1.17 (1.02, 1.35)	0.024
Model 3					
Cumulative Cystatin C groups	Q1				
Q2		0.86 (0.47, 1.58)	0.637	1.07 (0.83, 1.19)	0.600
Q3		0.76 (0.41, 1.41)	0.386	0.97 (0.74, 1.28)	0.853
Q4		0.78 (0.42, 1.45)	0.435	1.26 (0.96, 1.67)	0.102
Model 4					
Cumulative Cystatin C categories	Level 1				
Level 2		1.31 (0.83, 2.08)	0.247	0.81 (0.66, 0.99)	0.040
Level 3		1.39 (0.67, 2.86)	0.374	2.12 (0.55, 8.18)	0.275

*Model 1, Model 2, and Model 3 were adjusted for age, education level, hematocrit, and HDL.

### Dose–response relationship

**Figure 5:**
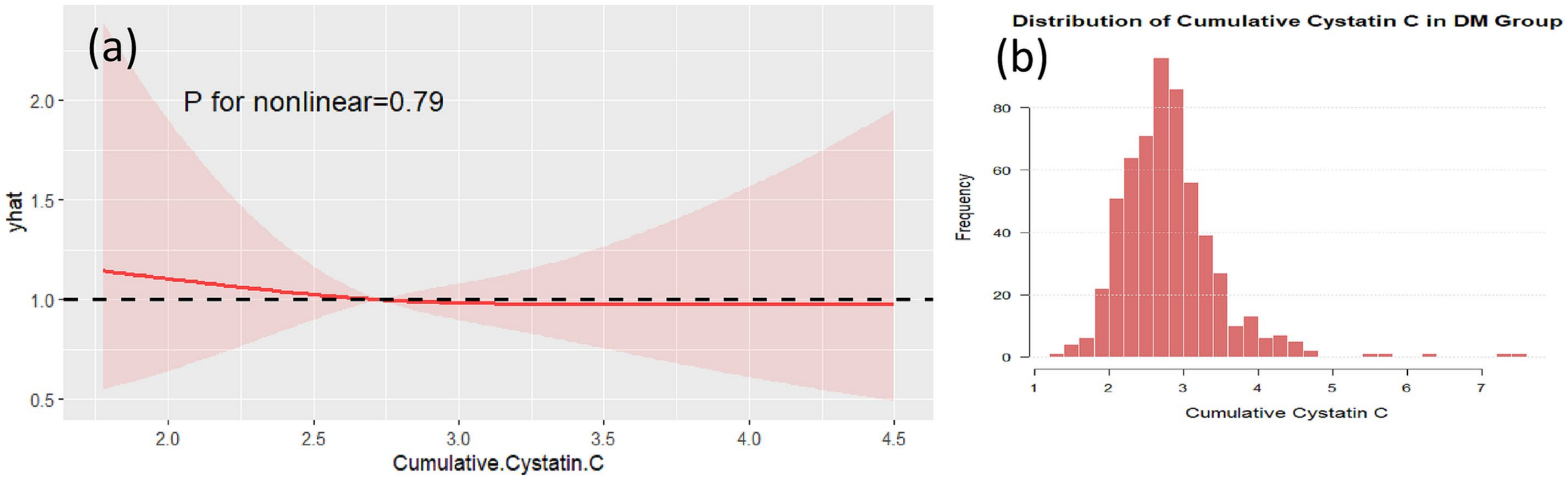
RCS analysis in the DM group. RCS plot showing no significant non-linear association between cumulative cystatin C and cognitive decline in the DM group (*p* = 0.79) **(a)**. The data distribution of the DM group is shown in **(b)**.

**Figure 6:**
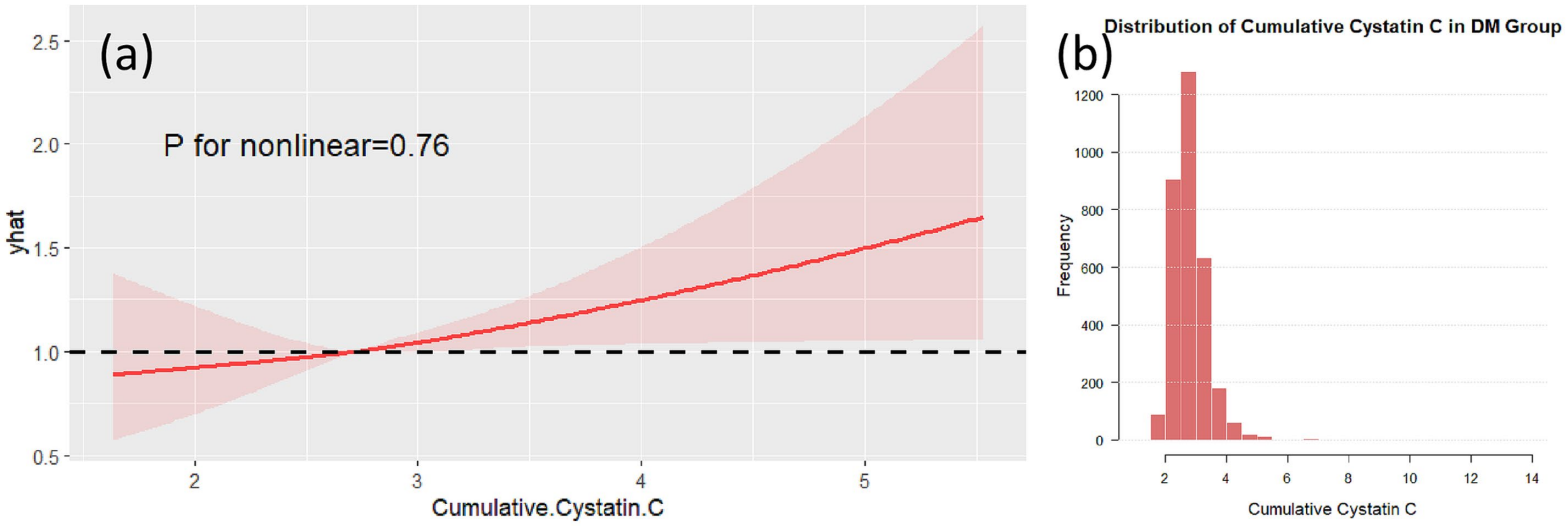
RCS analysis in the non-DM group. RCS plot showing no significant non-linear association between cumulative Cystatin C and cognitive decline in the non-DM group (*p* = 0.76) **(a)**. The data distribution of the non-DM group is shown in **(b)**.

Non-linear analysis of cumulative Cystatin C and cognitive impairment showed that, after adjusting for confounding factors, no non-linear associations were found between cumulative Cystatin C and cognitive impairment in either the DM group (Figure 5, *p* = 0.79) or the non-DM group (Figure 6, *p* = 0.76). Figures 5, 6, respectively, demonstrate the nonlinear analysis results (Figures 5a, 6a) and data distribution histograms (Figures 5b, 6b) for the DM group and non-DM group.

### Subgroup analysis

**Figure 7:**
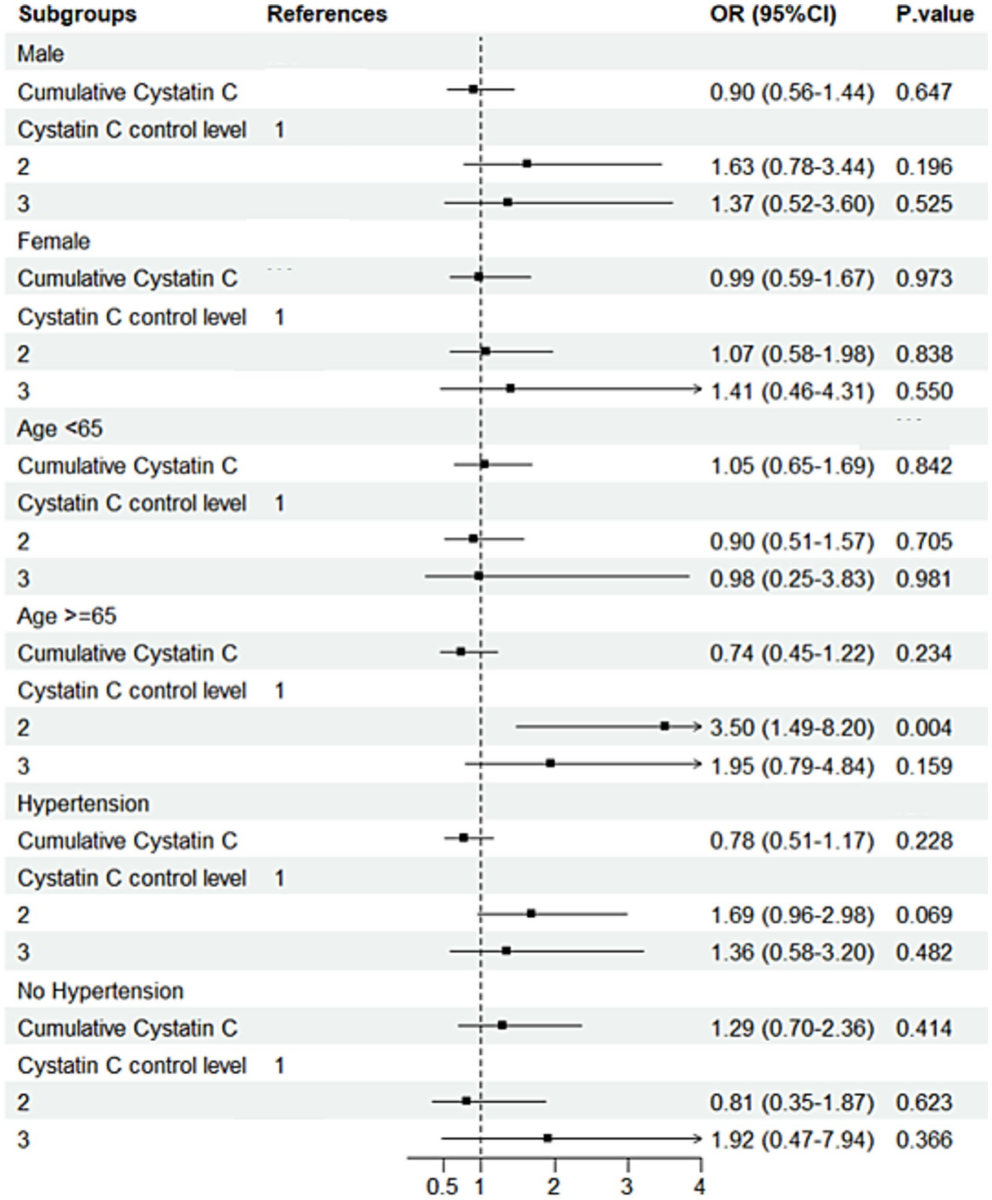
Subgroup analysis forest plot for the DM group. Forest plot showing subgroup analysis results for cognitive decline risk in the DM group.

**Figure 8:**
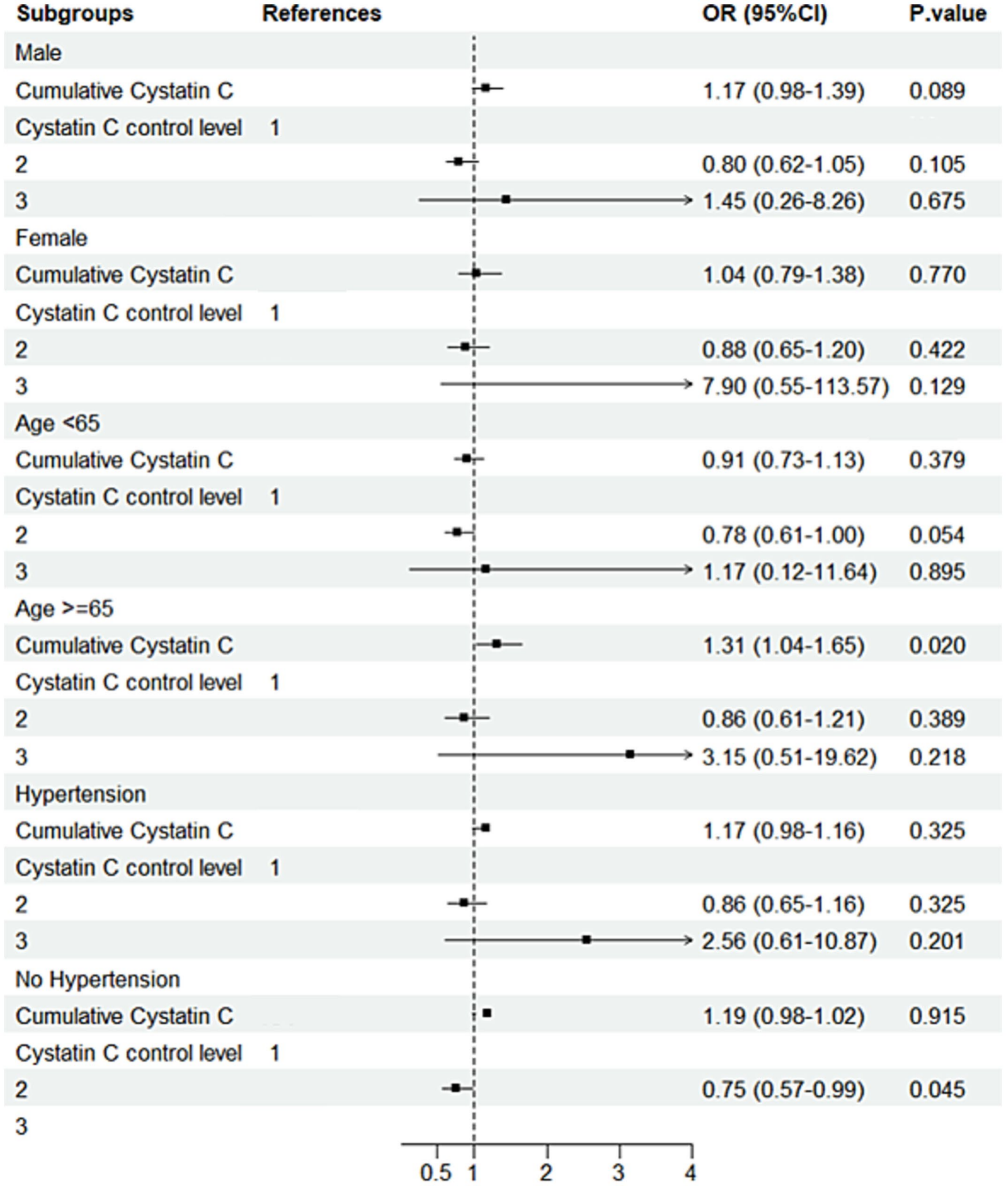
Subgroup analysis forest plot for the non-DM group. Forest plot showing subgroup analysis results for cognitive decline risk in the non-DM group.

Subgroup analysis results indicated that, in individuals with diabetes and aged ≥65 years, compared to Cystatin C Level 1, Level 2 was associated with a 250% increased risk of cognitive decline (OR = 3.50, 95% CI: 1.49–8.20, *p* = 0.004; Figure 7). Further analysis of baseline characteristics in this subgroup revealed that the mean age was 70.47 years, with a mean creatinine level of 0.84 mg/dL. Among the participants, 124 (76.1%) had hypertension, and 8 (4.9%) had a history of stroke. In non-diabetic individuals aged ≥65 years, each one-unit increase in Cystatin C was associated with a 31% increased risk of cognitive decline (OR = 1.31, 95% CI: 1.04–1.65, *p* = 0.020). In non-diabetic and non-hypertensive individuals, compared to Cystatin C Level 1, Level 2 was associated with a 25% decreased risk of cognitive decline (OR = 0.75; Figure 8). The number of subjects in each subgroup and the number of events in each trajectory category are listed in Supplementary Table 3.

### Mediation analysis

**Figure 9:**
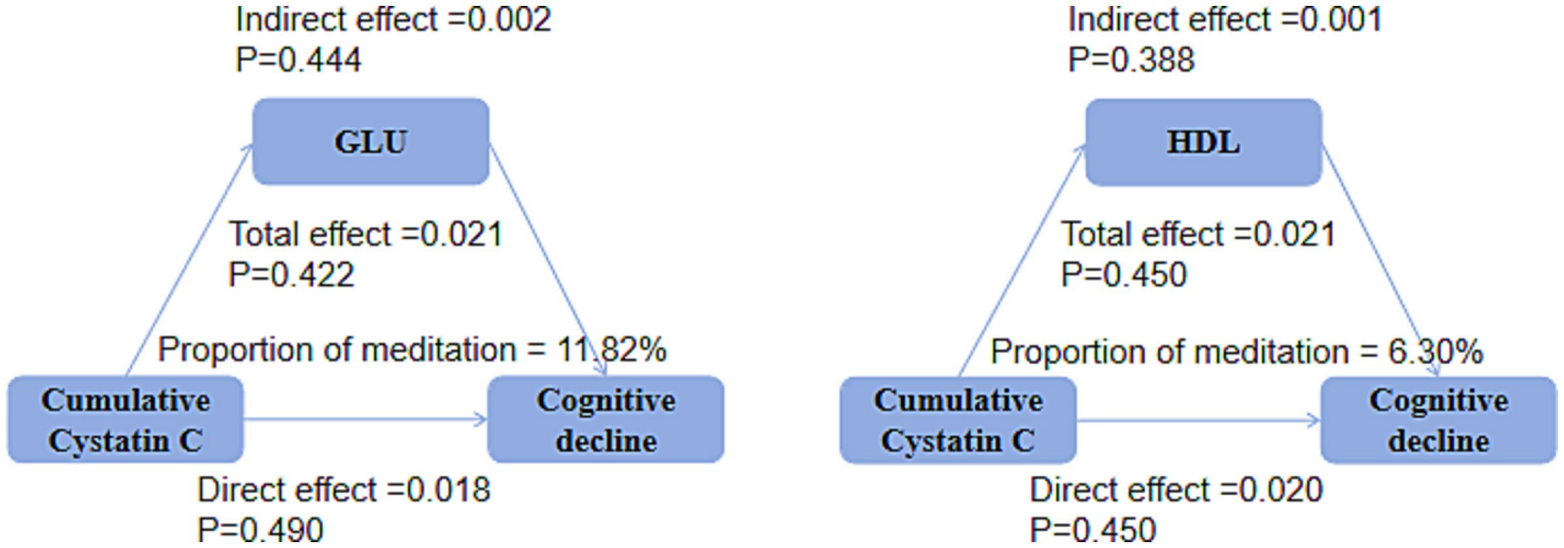
Mediation analysis in the DM groups. Mediation analysis indicating no significant effects of glucose or HDL on the relationship between cumulative cystatin C and cognitive decline in either the DM groups (*P*0.05).

**Figure 10:**
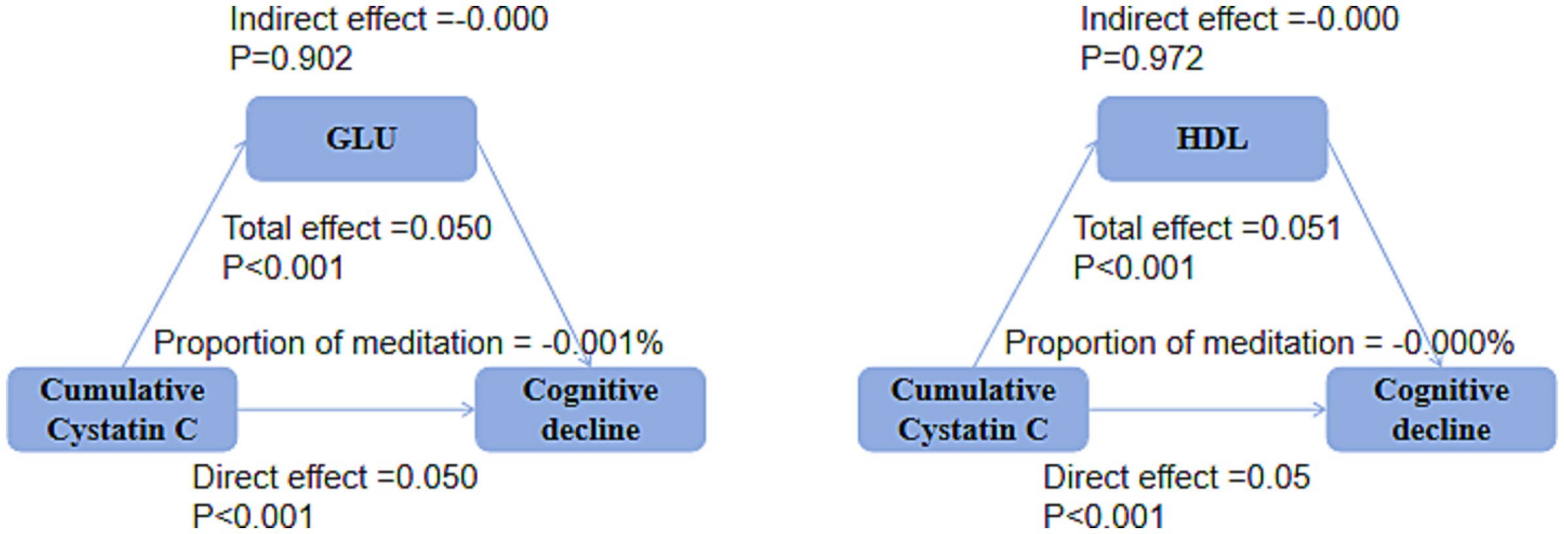
Mediation analysis in the non-DM groups. Mediation analysis indicating no significant effects of glucose or HDL on the relationship between cumulative Cystatin C and cognitive decline in either the non-DM groups (*p* > 0.05).

Mediation analysis showed that, in both the DM group (Figure 9) and the non-DM group (Figure 10), fasting glucose levels and HDL did not mediate the relationship between cumulative Cystatin C and cognitive decline (all *p* > 0.05).

## Discussion

The present study, based on a nationwide cohort dataset, aims to explore the association between the long-term trends of Cystatin C and cognitive decline in populations with different states of glucose metabolism. Our findings indicate that, in non-diabetic individuals, maintaining a low level of Cystatin C is associated with a reduced risk of cognitive decline. Conversely, in older individuals with diabetes, maintaining a low level of Cystatin C is associated with an increased risk of cognitive decline. These results suggest that differential management strategies for Cystatin C are needed for populations with different glucose metabolic statuses to reduce the risk of cognitive decline.

The relationship between Cystatin C levels and cognitive function has long been a subject of significant controversy. Cystatin C, with its extensive biological functions, including bone resorption, inhibition of extracellular cysteine protease activity, modulation of inflammatory responses, and stimulation of proliferation in glomerular mesangial cells and fibroblasts (17–19), has been widely investigated. The Health ABC study revealed that higher levels of Cystatin C are associated with lower cognitive function scores and an increased risk of cognitive impairment (7). Slinin et al. found in their study on elderly women that there is a U-shaped relationship between Cystatin C concentration and cognitive function after 10 years, but this association disappeared after adjusting for confounding factors (9). However, a study on stroke survivors showed that in patients with normal renal function, Cystatin C acts as a protective factor against post-stroke cognitive impairment, with an inverse correlation between Cystatin C levels and the risk of cognitive impairment (20). Previous studies have demonstrated that Cystatin C can bind to soluble amyloid-*β* peptides and inhibit amyloid deposition in the brains of amyloid precursor protein (APP) transgenic mice, thereby reducing the risk of dementia (21). Cystatin C can also help prevent cognitive decline by modulating immune responses and reducing oxidative stress (22). Cystatin C has specific, saturable, and high-affinity binding to Abeta (1–42) and Abeta (1–40), thereby inhibiting the formation of Abeta fibrils (17).

This study is the first to stratify analyses based on diabetes and non-diabetes. The results show that in non-diabetic individuals, maintaining a low level of Cystatin C is associated with a lower risk of cognitive decline, while in older individuals with diabetes, maintaining lower levels of cystatin C is associated with cognitive decline. This may be due to the different metabolic patterns and pathological processes in diabetic and non-diabetic individuals. In non-diabetic populations, lower cystatin C levels typically reflect intact glomerular filtration function and the autophagy-lysosomal system’s ability to clear Aβ and phosphorylated tau proteins. Its inhibition of cysteine protease activity also limits microglial overactivation, reduces oxidative stress and synaptic loss, thereby lowering the risk of cognitive decline. Conversely, persistently low cystatin C levels in elderly individuals with diabetes may indicate a “decompensated” state: hyperglycemic toxicity, advanced glycation end products, and glomerular hyperfiltration accelerate tubular protein loss, leading to “dilutional” reduction of cystatin C accompanied by protein-energy expenditure. Insulin resistance further suppresses its gene transcription, upregulates tissue protease B/S activity, and shifts the protease-antiprotease balance toward extracellular matrix degradation and blood–brain barrier leakage, with its protective effects offset by diabetes-driven proteolysis and neuroinflammation. Hypertension and diabetes mellitus often coexist, and both affect cognition through mechanisms such as endothelial dysfunction, microvascular damage, and chronic inflammation. If stratified solely based on “non-DM,” a significant number of hypertensive patients may still be confounded, with their cognitive decline potentially driven by blood pressure-related pathways. Therefore, we further constructed a subgroup of “non-DM and non-HPN” to approximate a “relatively pure” population, thereby reducing confounding from glucose metabolism or blood pressure-related cerebrovascular lesions and enabling the isolation of independent biomarker effects. We found that the protective effect of low Cystatin-C was only significant in this “double-negative” subgroup, suggesting that its cognitive benefits may be offset by the presence of DM or HPN.

In elderly patients with diabetes, the low cystatin C trajectory is associated with cognitive decline, which may also reflect reverse causality or confounding factors. Firstly, early neurodegeneration or systemic inflammation can reduce the protein synthesis capacity of hepatocytes and renal tubular epithelial cells, directly lowering cystatin C concentrations. Secondly, low cystatin C levels are often accompanied by muscle mass loss, nutritional deficiencies, or sarcopenia, which are themselves predictors of cognitive deterioration. Additionally, persistent low-grade inflammation, oxidative stress, and vascular endothelial damage in diabetic conditions may simultaneously inhibit cystatin C production and accelerate brain structural damage, resulting in a “low-value-high-risk” phenotype. Therefore, lower cystatin C levels in this population are more likely to be a “consequence” rather than a “cause” of disease progression. Future studies should further clarify the causal direction through longitudinal proteomics, muscle mass assessment, and inflammatory biomarkers. We have adjusted for hematocrit in the multivariate model, which to some extent reflects the nutritional or metabolic status of the subjects; however, this may not directly quantify muscle mass or protein-energy malnutrition. Therefore, the association between low cystatin C and cognitive decline may still be influenced by residual confounding from sarcopenia/weakness factors, and future validation may require more direct measures of muscle mass, such as grip strength, bioelectrical impedance, or serum cystatin levels. Further studies are needed to verify the conclusions of this study and to further explore the underlying associations.

There is a close relationship between glucose metabolism and cognitive function, but the role of glucose metabolism in the relationship between Cystatin C and cognitive function is still unclear. A study on Asian populations showed that Cystatin C levels are highly correlated with the number of metabolic syndrome components (23). In addition, serum Cystatin C levels are positively correlated with the incidence of type 2 diabetes after 15 years of follow-up (24). Li et al. found that the association between Cystatin C and the risk of mild cognitive impairment (MCI) was only observed in diabetic populations, and that beta-cell function has a negative mediating effect in the relationship between Cystatin C and the risk of mild cognitive impairment (25). Type 2 diabetes and Alzheimer’s disease share a number of common pathological processes, such as amyloid-beta plaques, brain glucose metabolic disturbances, tau hyperphosphorylation, and inflammation (12–14). Studies have shown that a high-sugar diet is associated with worse cognitive performance and increased brain amyloid burden (26). Abnormal glucose homeostasis impairs cognitive function through a variety of pathways, such as microvascular damage, impaired glucose metabolism, and increased beta-amyloid deposition (25, 27, 28). Hyperglycemia and insulin resistance may lead to changes in the processing of amyloid precursor protein or beta-amyloid (29). In addition, hyperglycemia can lead to a state of slow brain metabolism, mainly localized near brain regions that are preferentially affected by Alzheimer’s disease (30–32). Type 2 diabetes may also increase the risk of Alzheimer’s disease by impairing brain glucose uptake/metabolism, thereby downregulating O-GlcNAcylation and promoting abnormal hyperphosphorylation of tau (33).

The results of this study show different association patterns between Cystatin C and cognitive function in diabetic and non-diabetic populations. The results of this study show that in non-diabetic populations, maintaining a low level of Cystatin C is associated with a lower risk of cognitive decline, while in older diabetic populations, maintaining a low level of Cystatin C increases the risk of cognitive decline. This may indicate significant differences in the impact of Cystatin C on cognitive function in different states of glucose metabolism. In non-diabetic populations, lower levels of Cystatin C may reflect better renal function and metabolic status, thereby protecting cognitive function. However, in older diabetic populations, maintaining a low level of Cystatin C may be related to renal function overcompensation or other metabolic disturbances, thereby increasing the risk of cognitive decline. In addition, diabetic patients may have more complex pathological processes, such as insulin resistance and chronic inflammation, which may interact with Cystatin C to further affect cognitive function. More studies are needed in the future to explore the complex associations between glucose metabolism, Cystatin C, and cognitive impairment.

### Limitations

Several limitations should be acknowledged in this study. First, our population data were entirely derived from China, which means that the study results may be influenced by regional and ethnic specificity. Therefore, further validation in other ethnic groups is needed to assess the generalizability and applicability of the findings. Second, some of the study results were based on self-reported data, which may introduce a certain degree of subjective bias, affecting the accuracy and reliability of the results. Third, the determinants of cognitive function are extensive, and considering the majority of confounding factors is a challenging task with a limited sample size. Although we adjusted for known confounding factors as much as possible in the analysis, there may still be unidentified or unmeasured confounding factors that could affect the results. Furthermore, although we included baseline blood glucose levels and glycated hemoglobin levels in the univariate correlation analysis to investigate blood glucose levels and glycemic control, no significant association was found between these two factors and cognitive decline. Therefore, we did not further adjust for these variables in the multivariate analysis. This limitation suggests that our results may not fully exclude the potential confounding effect of blood glucose levels on cognitive function. Future studies should consider more comprehensive glycemic control indicators, such as continuous glucose monitoring data, and more detailed evaluation of the specific impact of glycemic fluctuations on cognitive function. Finally, although appropriate cumulative exposure and cluster analysis were conducted under the current research conditions, future studies should incorporate more longitudinal repeated measurements (when feasible) to further elucidate the dynamic relationship between Cystatin C and cognitive function. This will help to more accurately assess the impact of long-term changes in Cystatin C on cognitive function.

## Conclusion

Based on a nationwide cohort dataset, this study is the first to investigate the association between long-term trends of Cystatin C and cognitive decline in populations with different glucose metabolic statuses. The findings indicate that, in non-diabetic individuals, maintaining a low level of Cystatin C is associated with a reduced risk of cognitive decline. Conversely, in older individuals with diabetes, maintaining a low level of Cystatin C is associated with an increased risk of cognitive decline. This study further elucidates the complex relationship between Cystatin C and cognitive function and highlights the need for differentiated preventive strategies in different glucose metabolic states to reduce the risk of cognitive decline. Future research can further explore the underlying biological mechanisms between Cystatin C and cognitive function, as well as the specific role of glucose metabolism, providing more evidence for the prevention and treatment of cognitive impairment.

## Data Availability

The original contributions presented in the study are included in the article/supplementary material, further inquiries can be directed to the corresponding authors.
